# Sequential Biorefining of Bioactive Compounds of High Functional Value from Calafate Pomace (*Berberis microphylla*) Using Supercritical CO_2_ and Pressurized Liquids

**DOI:** 10.3390/antiox12020323

**Published:** 2023-01-30

**Authors:** Jaime Ortiz-Viedma, José M. Bastias-Montes, Cielo Char, Camila Vega, Alejandra Quintriqueo, Manuela Gallón-Bedoya, Marcos Flores, José M. Aguilera, José M. Miranda, Jorge Barros-Velázquez

**Affiliations:** 1 Departamento de Ciencia de los Alimentos y Tecnología Química, Facultad de Ciencias Químicas y Farmacéuticas, Universidad de Chile, Dr. Carlos Lorca 964, Santiago 8320000, Chile; 2 Departamento de Ingeniería en Alimentos, Universidad del Bio-Bio, Avda Andrés Bello 720, Chillan 3800708, Chile; 3 Facultad de Ciencias Agrarias, Universidad Nacional de Colombia, Sede Medellín, Medellín 050034, Colombia; 4 Departamento de Ciencias Básicas, Facultad de Ciencias, Universidad Santo Tomás, Talca 3460000, Chile; 5 Departamento de Ingeniería Química y Bioprocesos, Universidad Católica de Chile, V. Mackenna 3860, Santiago 8940000, Chile; 6 Departamento de Química Analítica, Nutrición y Bromatología, Facultad de Veterinaria, Universidad de Santiago de Compostela, 27002 Lugo, Spain

**Keywords:** antioxidant capacity, bioactive components, biorefinery, calafate residual pomace, supercritical extraction, pressurized solvents, functional ingredients

## Abstract

A biorefinery process was developed for a freeze-dried pomace of calafate berries (*Berberis microphylla*). The process consisted of extraction of lipophilic components with supercritical CO_2_ (scCO_2_) and subsequent extraction of the residue with a pressurized mixture of ethanol/water (1:1 *v*/*v*). scCO_2_ extracted oil from the pomace, while pressurized liquid extraction generated a crude extract rich in phenols and a residue rich in fiber, proteins and minerals. Response surface analysis of scCO_2_ extraction suggested optimal conditions of 60 °C, 358.5 bar and 144.6 min to obtain a lipid extract yield of 11.15% (d.w.). The dark yellow oil extract contained a good ratio of ω6/ω3 fatty acids (1:1.2), provitamin E tocopherols (406.6 mg/kg), and a peroxide index of 8.6 meq O_2_/kg. Pressurized liquid extraction generated a polar extract with good phenolic content (33 mg gallic acid equivalents /g d.w.), anthocyanins (8 mg/g) and antioxidant capacity (2,2-diphenyl-1-picrylhydrazyl test = 25 µg/mL and antioxidant activity = 63 µM Te/g). The extraction kinetics of oil by scCO_2_ and phenolic compounds were optimally adjusted to the spline model (R^2^ = 0.989 and R^2^ = 0.999, respectively). The solid extracted residue presented a fiber content close to cereals (56.4% d.w.) and acceptable values of proteins (29.6% d.w.) and minerals (14.1% d.w.). These eco-friendly processes valorize calafate pomace as a source of ingredients for formulation of healthy foods, nutraceuticals and nutritional supplements.

## 1. Introduction

Berries are an important source of bioactive secondary metabolites such as dietary antioxidants and nutrients such as fiber and polyunsaturated fatty acids, associated with health benefits [1,2]. In addition, berries, due to their high-water content (>80%), are low in calories [3]. Bioactive compounds found in berries, classified as phenolic acids, stilbenes, flavonoids, tannins and lignans, vary according to genetic factors, environmental conditions, stages of maturity, harvest time, postharvest handling and storage conditions of the fruit [3]. In recent years, the term “superfruit” has gained popularity and has been used to promote the health benefits of “exotic fruits” that grow wild under certain climatic conditions or are cultivated on a small scale by local people [4]. This is the case for acai (*Euterpe oleracea*), acerola (*Malpighia emarginata*), camu-camu (*Myrciaria dubia*), goji (*Lycium barbarum*) and blueberries (*Vaccinium* sect. *Cyanococcus*), among others [4]. Patagonian berries maqui (*Aristotelia chilensis*), murta (*Ugni molinae Tuscz*) and calafate (*Berberis microphylla*) are considered “super fruits” due to their high content of phenolic compounds, including several anthocyanins [5,6]. Calafate is a shrub that produces a dark-skinned berry and grows extensively in southern Chilean and Argentine Patagonia [7]. Several studies have shown the strong antioxidant potential of the calafate fruit due to the content of anthocyanins, polyphenols and hydroxycinnamic acids [5,7,8,9,10,11,12,13,14,15,16]. Brito et al. [8] reported that calafate berries had a higher content of anthocyanins than six other berries. Ramirez et al. [5] compared the antioxidant activity of six Chilean berries and determined that calafate inhibited lipid peroxidation in human erythrocytes, mitigating the spread of oxidative stress [5]. Other work [6] found 18 types of anthocyanins in an extract of calafate, exceeding the levels described for maqui and murtilla [6]. Additionally, Speisky et al. [16] determined the antioxidant activity (oxygen radical absorbance capacity (ORAC)) of more than 120 species/varieties of fruits and found that calafate was the fruit with the greatest antioxidant potential. Associated with its high antioxidant capacity and content of phenolic compounds, mainly anthocyanins, calafate has been reported to exhibit anti-inflammatory [9,12,13,15], antiproliferative [9], vasodilatory [17] and anti-atherosclerotic effects on tumor cells [18].

In addition, calafate extracts have been shown to restore insulin-induced protein kinase B (AKT) phosphorylation and glucose tolerance in a diet-induced obesity model using mice [15] and to inhibit the enzyme α-glucosidase, affecting carbohydrate digestion and thus controlling postprandial hyperglycemia. The authors determined that the administration of calafate extract increased the concentration of 16 antioxidant phenolic acids in mice plasma [7,10].

Calafate berries can be consumed fresh or processed in products such as jellies, juices, jams and alcoholic beverages [9,11]. When processing berries, byproducts are generated that can be composted, used as components in the formulation of animal feed or discarded in landfills [19], losing a considerable amount of nutrients and phytochemicals [20]. To recover valuable compounds and valorize byproducts, zero-waste green technologies have been applied based on biorefining. Supercritical fluid extraction (SFE) is a well-known technology, with several applications in foods. scCO_2_ is considered a green technology since it has minor impacts on the environment and CO_2_ is a solvent generally recognized as safe. scCO_2_ has been proposed for the extraction of many bioactive compounds from plant material such as phenols, coumarins and alkaloids, among others [21,22]. CO_2_ above its critical temperature and pressure makes compound recovery very easy and provides a solvent-free analysis [23]. scCO_2_ extraction is efficient for the complete recovery of neutral lipids from various plant raw materials depending on their particle size [24] as well as constituents of microalgae. Extracted bioactive compounds find application in the nutraceutical, food and energy industries, among others [25].

Pressurized solvents or enzymatic methods have also shown promising results in extraction from rowanberry byproducts [19], blackcurrant [1], blackberry, lingonberry [20], raspberry [25] and cranberry [26]. Supercritical extraction with carbon dioxide (scCO_2_) and pressurized solvents utilizes nontoxic, relatively inexpensive, readily available, environmentally friendly and food-grade safe (GRAS) solvents [1,20]. In scCO_2_, CO_2_ penetrates solid particles faster than liquid solvents, and extraction can be carried out at low temperatures, maintaining the properties of heat-sensitive compounds [1]. In pressurized solvent technology, high pressure keeps solvents in the liquid phase, and if temperature is applied, contact between solvent and matrix can be maximized by increasing diffusion rates for mass transfer to the solvent [27]. In berries, the seeds and the skin contain high levels of polyphenolic compounds, fiber, lipophilic compounds and minerals [26]. By combining extraction technologies, it has been possible to obtain extracts with different compositions. scCO_2_ extraction is used for the extraction of lipophilic components [1]. On the other hand, in pressurized solvent extraction, the appropriate choice of a polar solvent allows the extraction, for example, of anthocyanins [28]. In the particular case of calafate, the extraction of bioactive compounds from byproducts or residues that include the seed and the skin using green technologies has not been extensively reported to date. Only small studies have been published, such as Ruiz et al. who studied the profile and concentration of flavonols in calafate skin, pulp and seed, reporting lower concentrations of flavonol in seed compared to pulp and peel [29]. Additionally, Mazzuca et al. described the fatty acid profile of seed oil from two species of Argentine calafate (*Berberis buxifolia* and *Berberis heterophylla*), where linoleic and oleic acids predominated [30]. Similar results were reported by Olivares-Caro et al.; therefore, the components of the calafate byproduct represent a potential source of functional ingredients for food and other uses in nutraceuticals, cosmetics and pharmaceuticals [18].

The objective of this study was to design a biorefining process for bioactive components which could constitute a source of functional ingredients applicable in the development of healthy foods, nutraceuticals and nutritional supplements from waste (pomace) from the powdered calafate industry. The process consisted of two sequential extractions applied to the same sample. To obtain lipophilic extracts, extraction with scCO_2_ was first applied, and later, to obtain hydrophilic extracts, accelerated hydroalcoholic extraction with pressurized liquid extraction (PLE) was applied. The scCO_2_ extraction was optimized by the response surface method (RSM). Furthermore, both scCO_2_ and PLE extraction kinetics were modeled by the spline method described by Jesus et al. [31]. Fatty acid profiles, tocopherols and physicochemical properties were determined for the lipophilic extracts. Total phenols, anthocyanins and antioxidant capacity were determined in the hydrophilic extract. Finally, the residual solid from the two extractions was converted into flour, and its nutritional value was determined to define its use as a food ingredient rich in fiber, minerals and proteins.

## 2. Materials and Methods

### 2.1. Raw Material

The raw material used corresponded to dry pomace (6.65% *w*/*w* moisture) of calafate (*Berberis microphylla*) composed of seeds, skins and fruit pulp, which was harvested in October 2021 and provided by Patagonia Superfruits S.A. (XI Region of Aysén, Chile). The average particle size of the residual calafate pomace (CR) was 589 ± 35 µm, obtained by sieving in an automatic shaker (Erweka-Apparatebau GMBH 6056, Heusenstamm, Germany).

### 2.2. Biorefining of Calafate Pomace

**Figure 1:**
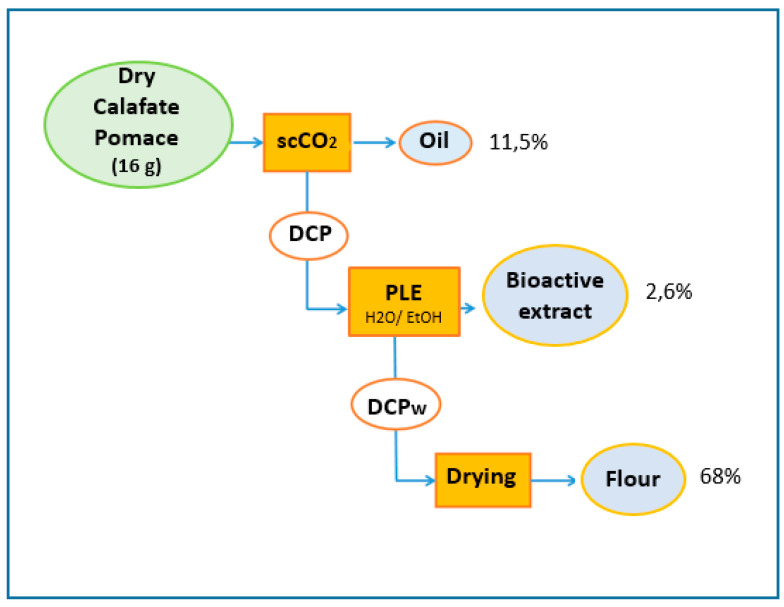
Scheme of the sequential biorefinery extraction by scCO_2_ and pressurized liquid extraction (PLE) of calafate pomace to obtain oil, bioactive extract and flour rich in fiber, protein and minerals. DCP: defatted calafate pomace (waste), DCPw: wet defatted calafate pomace.

The biorefining process began with extraction of the oil using scCO_2_ and then proceeded to extraction of the defatted calafate product (DCP) with a pressurized ethanol:water mixture (1:1 *v*/*v*) to obtain a bioactive extract high in polyphenolic components. The wet residue of DCP (DCPw) was dried in an oven at 30 °C for 30 min to obtain a flour rich in fiber, protein and minerals (Figure 1).

### 2.3. Supercritical Extraction with Carbon Dioxide

The supercritical extraction process was performed as described by Basegmez et al. [1], with some modifications using a Speed SFE-2 model 7071 supercritical extractor (Applied Separations, Allentown, PA, USA) coupled to a chiller system (F-200, Julabo USA Inc., Allentown, PA, USA). The 50 mL extraction cell was loaded with 16 g of calafate pomace. Liquid CO_2_ (purity 99.99%, Indura SA, Santiago, Chile) was used at a superficial speed of 1 mm/s. The temperature, pressure and extraction time were programmed as established in the experimental design.

#### Experimental Design for Extraction with scCO_2_

Optimal conditions of temperature, pressure and time for oil extraction from calafate pomace were determined by response surface methodology (RSM) following a Box-Behnken design at three levels of the independent variables: extraction temperature (Te: 30, 45, 60 °C), extraction pressure (P: 300, 350, 400 bar) and extraction time (Ti: 60, 105, 150 min). A total of 15 experiments were performed. Oil yield was considered a dependent variable based on the following polynomial equation of second order.

(1) $$Y=\beta_{0}+\beta_{A}A+\beta_{B}B+\beta_{C}C+\beta_{AB}AB+\beta_{AC}AC+\beta_{BC}BC+\beta_{A^{2}}A^{2}+\beta_{B^{2}}B^{2}+\beta_{C^{2}}C^{2}$$

where β_0_ is the intercept; β_A_, β_B_ and β_C_ are the coefficients of the factors; β_AB_, β_AC_ and β_BC_ are the coefficients of interactions between factors; and β_A_2, β_B_^2^ and β_C_^2^ are the coefficients of the double interactions. The model was determined using the lack of fit method and the coefficient of determination R^2^.

### 2.4. Pressurized Liquid Extraction

Extraction with PLE was performed as described by Basegmez et al. [1] with some modifications, using Dionex ASE^®^300 equipment (Thermo Fisher Scientific, Waltham, MA, USA) with adjustment and control of pressure, temperature and time. A total of 4.1 + 0.1 g of DCP from the supercritical extraction process was mixed with 1 g of celite in an extraction cell. The PLE extraction process was carried out with the addition of 40 mL of ethanol/water mixture (1:1 *v*/*v*) per cycle. The number of extraction cycles was evaluated by determining the total phenol content measured by the Folin–Ciocalteu methodology at the end of each cycle. Each cycle of 5 min was carried out at a standard pressure of 1500 psi and 25 °C. 

### 2.5. Modeling of Extraction Kinetics

#### 2.5.1. Modeling of the Extraction Kinetics with scCO_2_

Extraction curves of oil with scCO_2_ and phenolic extracts with PLE were fitted to the spline model described by Jesus et al. [31] using MATLAB R2020b software (CA, USA). The model describes three consecutive stages defined by the rate of extraction and associated with the release mechanisms. The first stage corresponds to a constant extraction rate (cer) described by convection, the second to a falling extraction rate (fer) defined by convection and diffusion, and the third to a period controlled by diffusion (dc). Each extraction stage is described by straight lines represented by Equations (2)–(4).

Y =b0 + b1 ∗ t   for t ≤ tcer(2)

Y = b0 − tcer ∗ b2 + (b1 + b2) ∗ t    for tcer < t ≤ tfer(3)

Y = b0 − tcer ∗ b2 − tfer ∗ b3 + (b1 + b2 + b3) ∗ t       for tfer < t(4)

where Y corresponds to the oil extraction yield per scCO_2_ bi (i = 0, 1, 2, 3) are the linear coefficients of each stage; tcer is the time for constant extraction rate; and tfer is the time period for falling extraction rate.

#### 2.5.2. Modeling of the Extraction Kinetics with PLE

Likewise, the modeling of the hydrophilic extraction kinetics by PLE uses the same Equations (2)–(4), where Y = yield expressed as total polyphenols of the hydrophilic extract.

### 2.6. Chemical Analysis

#### Nutritional Characterization

Calafate pomace and the residual product of the successive extractions by scCO_2_ and PLE were subjected to proximal analysis according to the official methods (AOAC, 2005) [32]. Moisture and ash content were determined by gravimetric methods, proteins by Kjeldahl, and lipids by the Soxhlet method with petroleum ether. Carbohydrates were determined by the Antrona colorimetric method after digestion with analytical grade perchloric acid. The colored complex formed between anthrone and the soluble sugars resulting from the hydrolysis of starch was read at 760 nm and expressed in g glucose/100 g [33].

### 2.7. Characterization of the Lipid Extract Obtained by scCO_2_

#### 2.7.1. Fatty Acid Profile

The fatty acid profile was determined by gas chromatography according to the official method Ce 2-66 AOCS (1998) [34] using an HP-5890 gas chromatograph (Hewlett-Packard, Palo Alto, CA, USA) with a 50 m long bpx-70 fused silica column, 0.25 µm film thickness and 0.25 mm internal diameter, with an Fid detector, and a split injection system, calibrated 90:10. The fatty acid methyl esters (FAMEs) obtained from Sigma-Aldrich (St. Louis, MO, USA) were prepared as follows: 100 mg of oil was mixed with 5 mL of 0.5 N sodium hydroxide solution in methanol and held in a thermoregulated bath for 5 min at 100 °C. Then, 5 mL of 12.5% boron trifluoride in methanol was added and heated for 3 min. Finally, 1.5 mL of petroleum ether and saturated sodium chloride solution were added. After gently shaking, the mixture was allowed to stand and promote phase separation to extract the FAME dissolved in petroleum ether.

#### 2.7.2. Tocols

Tocols composed of tocopherols and tocotrienols, were determined by high-performance liquid chromatography–fluorescence detector (HPLC-FL) as described in the official method Ce 8-89 AOCS (1998) [34]. A solution of 0.5% 2-propanol in hexane was used as the mobile phase. To prepare the sample, 100 mg of extracted oil was weighed into a 10 mL amber flask and brought to volume with hexane. Measurement runs were made for 35 min, injecting 80 µL of the sample. Tocols content was determined using α, β, γ and δ tocopherol and tocotrienol standard solutions (Calbiochem Merck, Darmstadt, Germany). The results were expressed in µg/g oil.

#### 2.7.3. Saponification Value

The saponification value (SV) was determined by the official method Cd 3-25 AOCS (1993) [35]. Briefly, 5 g of oil and 50 mL of alcoholic KOH solution were added to an Erlenmeyer flask connected to an air condenser to boil the mixture for 30 min. Once cool, the mixture was titrated with 0.5 M HCl using phenolphthalein. The results were expressed in mg KOH/g oil.

#### 2.7.4. Iodine Value

For the iodine value (YV), the Wijs method was used, as described in the official method Cd 1d-25 AOCS (1993) [35]. One hundred milligrams of completely dry and filtered lipid extract was dissolved in 15 mL of chloroform, 25 mL of Wijs iodide solution was added, and the samples were left to stand in the dark at 25 °C. Subsequently, 20 mL of KI solution was added, and the solution was titrated under constant stirring using a standard 0.1 M Na2S2O3 solution until the yellow color disappeared. Then, 1 to 2 mL of starch indicator solution was added, and the titration was continued until the blue color disappeared. The results were expressed in g I_2_/100 g oil.

#### 2.7.5. Free Fatty Acids

Free fatty acids (FFAs) were determined by titration according to the official method Ca 5a-40 AOCS (2009) [36] by mixing 10 g of the extracted oil with 1 mL of ethanol and 1 mL of diethyl ether neutralized with 0.1 N. Three drops of phenolphthalein were added, and titration was carried out with 0.1 N KOH until the color of the sample changed. The results were expressed as g oleic acid/100 g oil.

#### 2.7.6. Peroxide Value

The peroxide value (PV) was determined as described in the official method Cd 8-53 AOCS (2009) [36]. Five grams of oil and 30 mL of a 3:2 mixture of acetic acid:chloroform were added to a 250 mL Erlenmeyer flask containing 0.5 mL of saturated KI solution, and the sample was slowly titrated with a 0.1 M Na_2_S_2_O_3_ solution until the yellow color almost disappeared. Finally, 0.5 mL of a 1% starch solution was added, and the titration continued under vigorous stirring until all the I2 was released from the chloroform layer. The results were expressed as milli-equivalents of O_2_/kg oil.

#### 2.7.7. Oil Color Analysis

The determination of the oil color was carried out according to the official method Cc 13e-92 AOCS (2009) [36] using an oil tintometer (Lovibond^®^ brand PFXi-195/1, FL, USA). First, a cell was standardized to zero with no sample, and then the yellow standard was read, and finally, the oil sample. The equipment gives the color parameters of the oils measured in CIEL*a*b* coordinates where L*: is the luminosity (L* = 100, perfect white; L* = 0, black); a* measures redness (a* > 0, red; a* = 0, gray; a* < 0, green); and b* green-yellow tendency (b* > 0, yellow; b* = 0, gray; b* < 0, green).

### 2.8. Characterization of the Bioactive Extract Obtained by Pressurized Liquid Extraction

#### 2.8.1. Total Phenols

Determination of the total polyphenol content (TPC) was carried out by means of the Folin–Ciocalteu method, as described by Singleton and Rossi (1965) [37]. A total of 0.1 mL of the hydroalcoholic extract obtained by PLE was mixed with 4.9 mL of distilled water and 0.5 mL of Folin–Ciocalteu reagent. The sample was left to stand for 3 min, and 1.7 mL of 20% *w*/*v* anhydrous sodium carbonate solution was added. The absorbance of the sample was measured at 765 nm. The concentration of total phenols was determined by means of a calibration curve with gallic acid solutions between 50 and 800 µg/mL, and the results were expressed as mg gallic acid equivalents per dry weight of extract (mg GAE/g extract dw).

#### 2.8.2. 2,2-Diphenyl-1-Picrylhydrazyl Test

The antiradical capacity was measured by the 2,2-diphenyl-1-picrylhydrazyl (DPPH) test, as described by Brand-Williams et al. [38]. Briefly, 0.1 mL of extract and 3.9 mL of 1 mg/mL DPPH solution were added to a 15 mL tube. The solution was diluted to an absorbance range of 0.480 to 0.600. The mixture was left to stand in the dark for 30 min at room temperature. Subsequently, the absorbance at 517 nm was measured. Results were expressed as mg quercetin-3-rhamnoside per gram of dry extract (mgc3-o-glu/g dw).

The efficiency of the PLE extract as a free radical scavenger was determined by means of Equation (5).

(5) $$\%\;Discoloration=\frac{A_{c}-A_{m}}{A_{c}}\cdot 100$$

where *A_c_* is the absorbance of the control and *A_m_* is the absorbance of the sample.

#### 2.8.3. Oxygen Radical Absorbance Capacity Test

The antioxidant capacity was measured by the oxygen radical absorbance capacity (ORAC) method according to the methodology described by Huang et al. [39]. Twenty-five microliters of sample and 150 µL of fluorescein solution were incubated for 30 min at 37 °C. Subsequently, 25 µL of 4.6% AAPH solution (2,2′-azobis(2-methylpropionamidine) dihydrochloride) in phosphate buffer was added to initiate the reaction. The fluorescence intensity of the samples was recorded every 1 min using a 485 nm excitation filter with a 20 nm bandwidth and a 528 nm emission filter with a 20 nm bandwidth. The antioxidant capacity by ORAC was calculated by interpolation of the net area generated by the variation of the fluorescence intensity of the fluorescein of the samples in the linear regression of the areas under the curve generated by the kinetic variation of the fluorescein that was incubated with different concentrations of a Trolox standard (6-hydroxy-2,5,7,8-tetramethylchroman-2-carboxylic acid).

#### 2.8.4. Total Anthocyanins

The total anthocyanin content (TAC) was determined by the differential pH method proposed by Lee et al. [40] with some modifications. Extracts were diluted with the buffers KCl 0.025 M pH 1.0 and sodium acetate 0.4 M, pH 4.5, adjusting the pH of both solutions with HCl 0.01 M. Then, the absorbances of the diluted extracts were measured at 530 and 700 nm. These values were used in Equations (6) and (7) to obtain the anthocyanin content in equivalents of cyanidin-3-glucoside (EC-3G).

(6) $$A=(A_{530}-A_{700})\;pH1,0-\left(A_{530}-A_{700}\right)$$

(7) $$C-3G\left(\frac{mg}{ml}\right)=\frac{\left(A\cdot MW\cdot D\cdot 10^{3}\right)}{\left(\varepsilon \cdot I\right)}$$

where *A* is obtained from Equation (6); *MW* is the molecular weight of cyanidin-3-glucoside (449.2 g/mol); *D* is the extract dilution factor; 10^3^ is the conversion factor from grams to milligrams; *ε* is the molar extinction coefficient of cyanidin-3-glucoside; and *I* is the path length in 1 cm.

### 2.9. Statistical Analysis

The experiments and the characterization of the oil extracted by scCO_2_ and the hydroalcoholic extract obtained by PLE were carried out in triplicate. Results are expressed as means with standard deviation. For the response surface analysis, the analysis of variance (ANOVA) was considered with a confidence level of 95%, using the Statgraphics Centurion XVI software.

## 3. Results

### 3.1. Response Surface Methodology Optimization of Oil Extraction by scCO_2_

**Figure 2:**
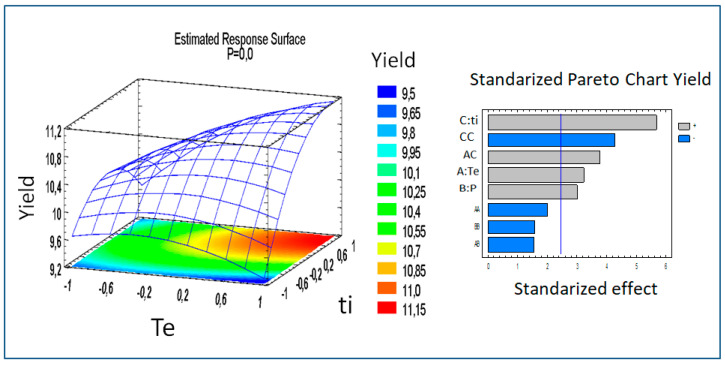
Response surface diagram of the experimental design of oil extraction by supercritical CO_2_ (indicating optimization zone in red) and Pareto diagram of interactions between variables. Te: extraction temperature, ti: extraction time, and P: pressure.

A graph of the response surface for the extraction process of oil from calafate’s pomace by means of scCO_2_ with the variables extraction temperature (te), extraction time (ti) and pressure (P) is shown in Figure 2. It is evident that the highest yields (11.5%) were presented for higher values of ti and te, while the lowest yields (9.5%) were for the entire range of te, when ti had the minimum values. According to the Pareto diagram (Figure 2), it is clearly shown that time, its quadratic interaction, temperature and pressure influence the extraction in a positive way, that is, these factors increase the yield.

The results of the ANOVA, carried out for the data obtained from the scCO_2_ of pomace of calafate oil, showed that the model expressed by Equation (8) had a determination coefficient (R^2^) of 93.9% and a nonsignificant lack of fit (*p* value > 0.05), which indicates that the model adequately represents the experimental data.

Yield = 10.72 + 0.2733 ∗ Te + 0.2557 ∗ P + 0.4817 ∗ ti−0.2501 ∗ Te2−0.1873 ∗ Te ∗ P + 0.4526 **∗** Te **∗** ti − 0.196 * P2−0.534 * ti2 (8)

The experimental optimization for the theoretical lipid extract yield of 11.15% dry weight (d.w.) yielded optimal extraction conditions with scCO_2_ at a temperature of 60 °C, 358.5 bar and 144.6 min.

The oil yield found in this study is higher than the 8.7% reported in supercritical extraction of cranberry pomace at temperatures of 53 °C, 158 min and 42.4 MPa [26]. On the other hand, for comparison, yields of 19.1, 14.6 and 6.6% oil have been obtained in washed, unwashed and dried berry pomaces from *Viburnum opulus L*., respectively, with optimal scCO_2_ conditions of 55–57 MPa, 120–131 min and 50 °C [41].

#### 3.1.1. Kinetic Model of Oil Extraction by Supercritical CO_2_

**Figure 3:**
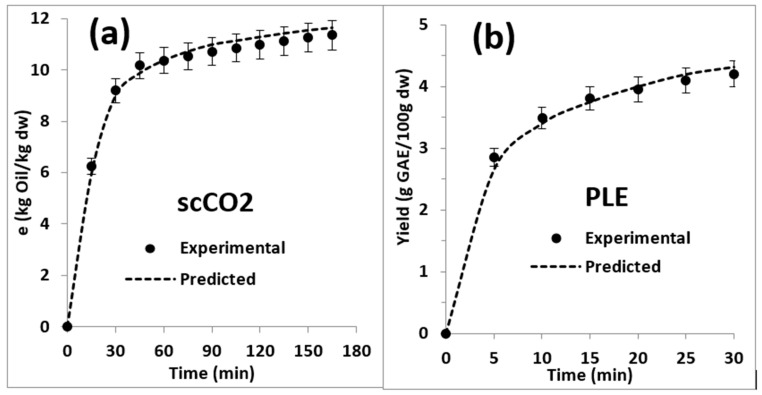
Experimental extraction curves fitted to the spline model. (**a**) Oil extraction curve with supercritical CO_2_ (358 bar and 60 °C). (**b**) Extraction of bioactive components from defatted calafate pomace (DCP) by pressurized liquid extraction (1500 psi and 25 °C) with an ethanol/water mixture (1:1 *v*/*v*).

The spline model suggests that the yield of oil extraction from calafate pomace tends to increase over time (see Figure 3), although the greatest changes occurred between times 0 to 30 min, when the yield values went from 0 to 11%. These results are within the values for oil extraction of bilberry, blackcurrant, raspberry, highbush blueberry, lingonberry, cranberry, and American cranberry pomaces using scCO_2_ which vary between 12 and 18% [42]. 

At higher times, the performance remained practically constant and with slight increases that reached 11.15 after 165 min. The parameters tcer, tfer and Mcer defined by the fit to the spline model are shown in Table 1. The calculated values for the parameters describe very precisely the kinetics of the oil extraction curve for the two slope regions between 0–30 min and 30–165 min. The spline model is a simple strategy to model extraction curves [43]. Despite corresponding to an empirical model [43], the experimental and modeled curves manage to define three regions: a constant rate of extraction (cer) associated with mass transfer by convection, a decrease in the rate of extraction (fer) described by control of mass transfer by diffusion and convection, and a period where the extraction rate is controlled by diffusion (dc) [31]. This stage is identified by the mass transfer rate, defined as Mcer, corresponding to parameter b1 of the spline model equations. In general, the greatest extraction occurs at this stage, with values between 70 and 90% being observed [31]. In the extraction of calafate oil during the cer period, an 88.1% extraction was obtained. The tcer defines the minimum time that an extraction cycle must last, which in this case corresponded to 29.8 min. This allows us to reduce the extraction time and the consumption of solvents [44]. 

Subsequently, between 29.8 and 93.2 min, a second slope associated with the fer period is obtained, and at 93.2 min, a third slope corresponding to the dc period is obtained. The extraction rates in the periods fer (constant b2) and dc (constant b3) correspond to negative values, indicating that the diffusive extraction mechanisms were irrelevant, and the greatest contribution to the extraction yield is given by convection during the cer stage. This behavior has been studied in a similar way in elderberry, where three phases in the extraction kinetics were identified (Kitryte et al.) [20]. Similar results were reported by Tamkute et al. [26] for the extraction of cranberry pomace and for the graph of the extraction kinetics of currant pomace oil [1] and for several other fruits and berries in which it has been concluded that the rate of extraction is controlled by internal diffusion through the cell walls [45].

#### 3.1.2. Kinetic Model of Extraction of Bioactive Compounds by Pressurized Liquid Extraction 

Similar to the extraction curve of calafate oil by scCO_2_, the spline model adequately described (R^2^ > 0.9999) the extraction curve of water-soluble bioactive components of the DCP defatted residue PLE (Figure 1). Figure 3b shows the adjusted experimental curve for extraction by PLE based on the content of total phenols, which comprised six extraction cycles of 5 min each applied to the same sample of defatted calafate pomace (DCP). In the three initial PLE extraction cycles applied to the same DCP residue, the quantified total polyphenol content was 2.81, 0.56 and 0.27 g GAE/100 g d.w., respectively, giving an accumulated total of 3.37 g GAE/100 g bw. Because the content of total phenols in the extract after carrying out the sixth cycle only gave an accumulated 4.32 g GAE/100 g d.w., it could be concluded that it was enough to carry out the third extraction to obtain almost 85% of the extract rich in polyphenols. Modeling the experimental curve using the spline method allowed us to determine the three extraction stages defined by tcer and tfer (Table 1). For the optimal extraction time, defined at 5.7 min (tcer), an extraction yield of 69.1% was obtained, while at the end of the fer period (tfer = 17.5 min), the yield reached 90.9%. Similar results were obtained when extracting polyphenols from orange peel using PLE and fitting the three-stage spline model [44].

On the other hand, the extraction times and yield obtained with PLE were more efficient for DCP than those obtained by applying ultrasound assisted PLE to the extraction of phenolic compounds from passion fruit bagasse [43]. This would indicate that the operating conditions applied in the DCP residue (1500 psi = 10.3 MPa and 25 °C) facilitated entry of the solvent into the plant structure. In addition, the extraction rates of the period fer (constant b2) and dc (constant b3), associated with diffusive mechanisms, were not relevant in the total extraction since only with three cycles of 5 min of extraction, over 85% of the content of bioactive compounds expressed as total phenols of calafate pomace was obtained.

### 3.2. Chemical Characterization of the Products

#### 3.2.1. Characterization of Pomace Oil from Calafate

Table 2 shows the characterization of the oil extracted from calafate pomace under optimal operating conditions using the supercritical fluid methodology. Mainly monounsaturated and polyunsaturated fatty acids (MUFAS and PUFAS) were identified with a value of approximately 88.7% of the total methyl esters. Some benefits of the consumption of ω3 and ω6 have been studied, including the regulation of blood pressure, vascular function, control of tumor cell growth and help in neuronal development [46,47]. The content of MUFAS given mainly by oleic acid was very similar to that of blackberry and close to that of cranberry and goldenberry oils [48,49,50]. On the other hand, the total PUFAS content was very similar to that of goldenberry (*Physalis peruviana L*.) [50]. A good ratio of ω-6/ω-3 (1:1.2) was evidenced, with high values of α-linolenic acid (36.7 ± 0.2%) and linoleic acid (30.0 ± 0.1%).

The linoleic acid content of calafate pomace oil was lower compared to maqui, murta, blackberry, cape gooseberry and rosehip berries but higher in linolenic acid, which translates into an optimal ω-6/ω-3 [48,51,52,55]. On the other hand, it has been reported in oils from other fruit seeds from the southern zone of Chile, such as blackberry (6.3:1) and blueberry (1.5:1), a greater content of α-linolenic acid (18:3ω3) than in calafate pomace oil [57,58].

#### 3.2.2. Tocols Content

Table 3 presents the tocopherol and tocotrienol content of the oil extracted from calafate pomace by scCO_2_ and its comparison with the tocols of other oils obtained from different berry seeds. In calafate oil extracted by scCO_2_, a total content of 406.4 ppm tocols was determined, composed of 18, 31 and 50% α-tocopherol (α-T), α-tocotrienol (α-T3) and γ-tocotrienol (γ-T3), respectively.

**Figure 4:**
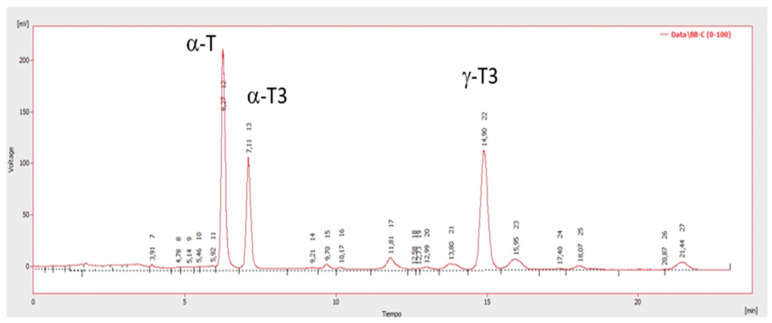
Chromatogram of the tocols present in the calafate sample by scCO_2_. α-T: Alfa-tocopherol, α-T3: alpha-tocotrienol, γ-T3: gamma-tocotrienol.

The oil obtained presents mainly γ-T3 tocols in its composition, which has been shown to have a higher antioxidant capacity than α-tocopherol at high temperatures when added to corn oil [61]. In vegetable oils, tocotrienols are scarce, particularly γ-T3, but cranberry and blueberry seed oils, as well as calafate oil, contain γ-T3. Calafate has a lower content of α-T and α-T3 compared to other berries, such as raspberry and maqui [48,59]. On the other hand, calafate pomace did not present γ-T, unlike maqui, and most other berries, including rosehip, where it is present at approximately 78% of the total content (1460 µg/g) of tocols [48,57]. The tocols present in the calafate oil extract by scCO_2_ can be observed in *n* the chromatogram of Figure 4.

#### 3.2.3. Quality Characteristics of Calafate Oil

The polyunsaturation degree of calafate pomace oil (Figure 2) with a YV = 159 was within the range reported by Firestone (2012) for blackberry, blackcurrant, blueberry and blackberry oils [48]. Strawberry’s YV (116 to 180) is justified by its high unsaturation provided by linoleic and α-linolenic acid [48]. The SV of 176 was representative of the average molecular weight of the fatty acids in the oil but lower than that of blueberry, raspberry [60] and other reported berry and fruit seed oils of similar composition [48,53,58,60]. This may be due to the analysis of the saponification value, which also includes the free fatty acids present in the oil. Regarding the quality characteristics of the lipid extract (Table 2), the values of the PV and FFA in calafate seed oil were 8.6 ± 0.4 meq O_2_/kg and 0.4 ± 0.1 mg/kg, respectively, which are within the range of fresh oils according to Chilean legislation [53] and values reported by other authors [48]. On the other hand, in addition to the oil present in berry seeds, high percentages of essential oils have been found in tissues from other parts of dark blue berries, including α-pinene (11.1%), linalool (11.6%), α-terpineol (15.7%), methyl eugenol (6.2%) and geraniol (3.7%) and in white berry oils, mirtenyl acetate (20.3%) [56].

#### 3.2.4. Color of Calafate Oil

The colors of the oils extracted by scCO_2_ were compared with those of other cold-pressed oils (Table 2). The L*a*b* color parameters indicated that the calafate pomace oil presented a dark yellow tone very similar to the color of cranberry but darker than that of maqui oil [51,54,62]. Possibly, the dark color of the oil was due to the migration of pigments such as carotenoids, chlorophylls, anthocyanins or other flavonoids from residues of skin and pulp. Components that could be present in the plant tissue that makes up pomace of calafate and influence the color of calafate seed oil during extraction with scCO_2_.

### 3.3. Characterization of the Phenolic Extracts Obtained by PLE

**Figure 5:**
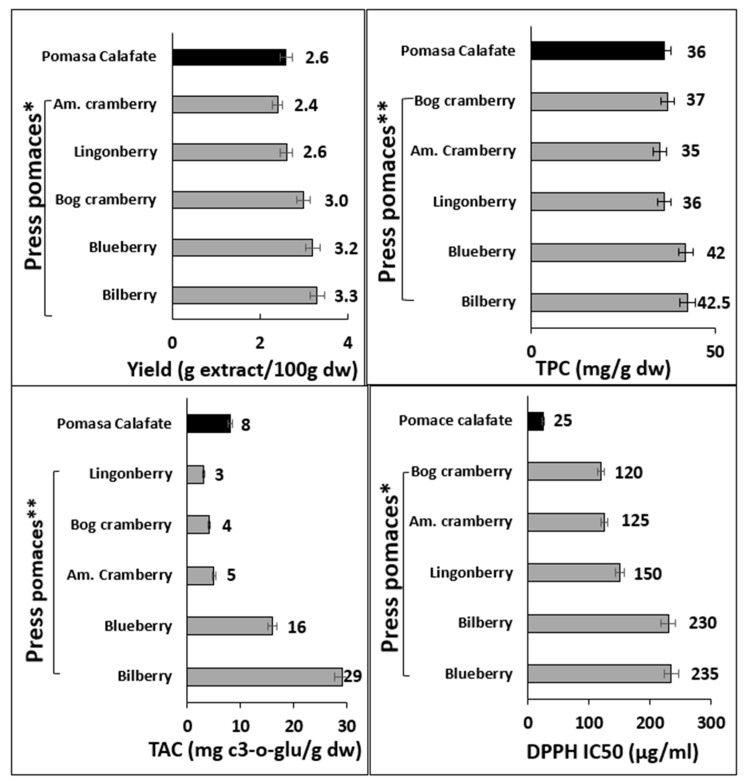
Yield and composition of the pomace extract from calafate obtained by pressurized liquid extraction and other hydroalcoholic extracts from the pomace of cold-pressed berries. * Muceniece et al. [65]; ** Klavins et al. [42].

Figure 5 shows the content of TPC, TAC, DPPH, and yield of calafate pomace compared with pomaces of other berries obtained by pressing [42,63,64]. The yield of the extracts obtained from calafate pomace obtained by PLE was close to the yield reported for extracts obtained by cold pressing of other berries but lower in the cases of blueberry, bog cranberry and bilberry. On the other hand, the phenol content of the calafate pomace extract obtained by PLE was similar to that of most berry pomaces, e.g., 80% of the blueberry and bilberry pomaces (Figure 5). The results indicated that the antiradical activity against DPPH of calafate pomace was considerably higher than that reported for most pressed berry pomaces [64].

These differences in extraction yield, polyphenol and anthocyanin content, and antioxidant capacity could be because calafate pomace is a residue obtained from sieving the freeze-dried fruit, in which the proportion of skin and pulp would be lower than that present in pomaces obtained by pressing berries. The lower content of pulp and skin would be reflected in a lower content of anthocyanins in the calafate pomace extract since these compounds are found mainly in the skin [3]. In blueberries, it has been observed that pressing and grinding prior to extraction break the epidermal tissue where the anthocyanins associated with the cell wall are found, increasing extraction [65]. Several studies have reported that the main anthocyanins in calafate are delphinidin-3-glucoside, petunidin-3-glucoside and malvidin-3-glucoside, with a smaller proportion of other polyphenols such as flavonols and flavan-3-noles [17,62]. On the other hand, it should be considered that some of the bioactive compounds of calafate pomace could have been dragged during the extraction of oil with scCO_2_.

**Figure 6:**
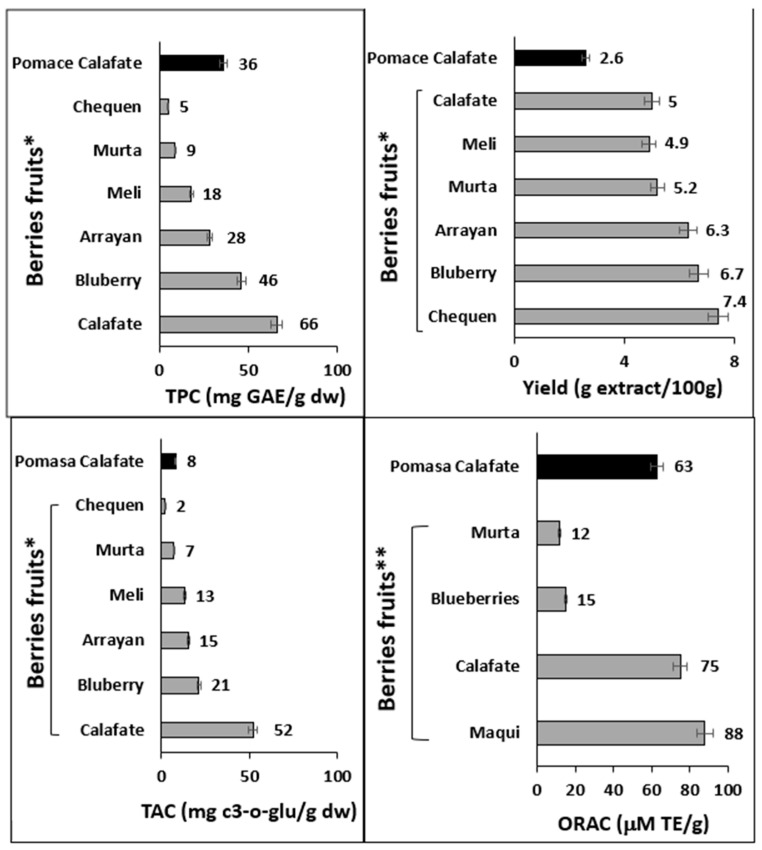
Oxygen radical absorbance capacity (ORAC), total polyphenols (TPC), total anthocyanin content (TAC), and yields of calafate pomace extracts obtained by pressurized liquid extraction compared with hydroalcoholic extracts from different Patagonian berry fruits. * Brito et al. [8] and ** Ruiz et al. [6].

Figure 6 compares the composition, antioxidant capacity and extraction yield of calafate pomace by PLE with the yield reported for hydroalcoholic extracts of fresh berry fruits [6,7]. The yield (2.6%) of the crude extract, obtained from calafate pomace by PLE, was close to half the yield obtained from the whole calafate fruit and at least a third of the yield of other fresh Chilean berries [7]. The anthocyanin content of the pomace was only 15% of that reported for the calafate fruit and was only higher than the content reported for murta and chequeen [8]. On the other hand, the antioxidant power given by the ORAC method for the calafate pomace extract was 85% with respect to that reported for the fresh calafate fruit and 71% of that presented by the fresh maqui fruit [6].

These results for the calafate pomace extract agree with the higher antioxidant capacity shown by calafate fruit extracts compared to other fruits and berries native to Patagonia [16]. Similar values have been reported for calafate extracts collected between December (2009) and February (2010) from different localities (Temuco, Lonquimay, Mañiguales, El Blanco) in Chilean Patagonia (Aysén, XI Region) that were in a range of 3.3 at 9.4 mg TE/g d.w. [11].

Other factors that influence the phenolic composition and antioxidant capacity of berries are variety, genetics, maturity, plant nutrition, harvest season and climate [3,5]. Climate is a fundamental factor considering the environmental stresses associated with Patagonia that would increase the synthesis of phenols [11].

On the other hand, it has been reported that the concentration of bioactive components in the seeds of berries is lower than that in the pulp and skin. One of the predominant flavonols found in calafate seed corresponds to quercetin-3-rhamnoside [6]. Although compounds derived from hydroxycinnamic acids have not been determined, the presence of delphinidin-3-glucoside, rutin and isorhamnetin rutinoside has been highlighted [10].

### 3.4. Nutritional Content of the Residual Flour of Calafate Pomace

**Figure 7:**
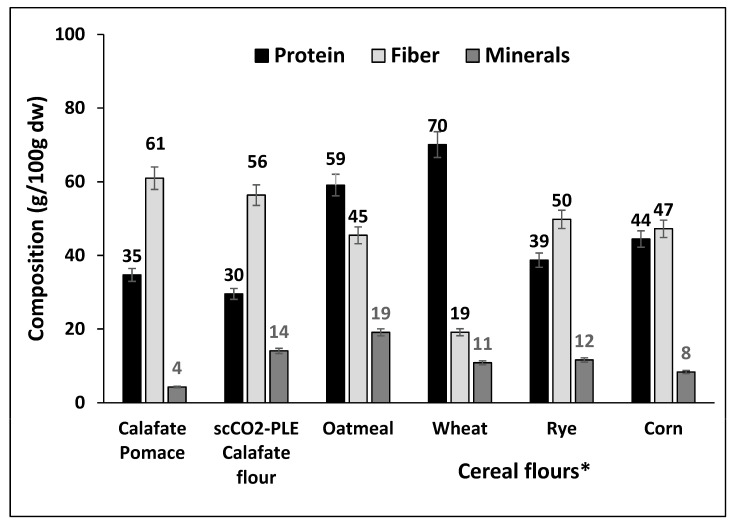
Content (d.w.) of protein, fiber and minerals in the fruit, pomace and calafate residual flour obtained after the scCO_2_-PLE extraction steps and its comparison with the nutritional composition of cereal flours [66,67]. * Corresponds to oatmeal, wheat, rye and corn [66,67].

Figure 7 shows the nutritional composition of calafate fruit (d.w.), the pomace and its residual flour (68 ± 1%) obtained after successive extraction with scCO_2_ and PLE. The protein content was quite high in the pomace before and after extraction by scCO_2_ and PLE, with values close to those of rice flour but lower than those of wheat, oats and corn [66,67]. However, the fiber contribution from calafate pomace and residual flour was higher than that provided by cereal flours [66,67]. The mineral content was higher than that of cereals (wheat, oats and corn) in the residual flour of calafate. In general, the nutritional contribution of the calafate flour was within the range of other flours, such as hazelnut, lentil, bean and soybean flours, used in formulations and nutritional supplements for human and animal nutrition [67].

Calafate has been praised for its large content of bioactive compounds. It is also notable for its high content of soluble solids, approximately 25–31° Brix, which is much higher than most other consumed berries [67,68]. Sugars present are largely fructose and glucose [6]. The beneficial high fiber content may prevent chronic noncommunicable diseases such as diabetes, colon cancer and hypercholesterolemia [68]. The protein and mineral contents of the residual calafate flour were relatively lower than those of cereal flours and other berries, such as murta [68].

## 4. Conclusions

Biorefining of calafate pomace using scCO_2_ and PLE produced lipidic and hydrophilic extracts and a residual flour-type supplement for human or animal nutrition. The optimal extraction conditions with scCO_2_ were 60 °C, 358.5 bar and 144.6 min, and a lipid extract yield of 11.15% (d.w.). The lipid extract presented a good content and ratio of ω-6/ω-3 fatty acids as well as tocopherol precursors of vitamin E. The oil exhibited good physical characteristics and a low oxidative state. This product could be used as a specialty ingredient in food formulations or as a nutraceutical. The hydroalcoholic extraction by PLE generated an extract with good phenolic content (80% of TPC) and antioxidant capacity comparable to that obtained in pressed pomace from other berries. The extraction kinetics from oil by scCO_2_ and phenolics by PLE were optimally adjusted to the spline model (R^2^ = 0.989 and R^2^ = 0.999, respectively). The final residual flour from the biorefinery process had a high fiber content and acceptable values of proteins and minerals, suitable for the development of nutritional supplements. This study verified the feasibility of using eco-friendly processes to recover oil, bioactive compounds and a high-fiber product from calafate pomace that may be used as ingredients in the development of healthy foods.

## Figures and Tables

**Table 1:** Spline model parameters for oil extraction by scCO_2_ and polyphenols by PLE from calafate.

Parameter	scCO_2_ Oil Extraction	PLE Polyphenol Extract
**t**_CER_ (min)	29.8	5.7
**t**_FER_ (min)	93.2	17.5
**b_0_**	6.17 × 10^−4^ (kg oil)	0 (g GAE)
**b_1_—M_CER_**	4.17 × 10^−4^ (kg oil/min)	0.526 (g GAE/min)
**b_2_—M_FER_**	−4.01 × 10^−4^ (kg oil/min)	−0.446 (g GAE/min)
**b_3_—M_dc_**	−4.90 × 10^−6^ (kg oil/min)	−0.048 (g GAE/min)
**error**	0.220	0.0096
**R^2^**	0.9890	0.9999

GAE: gallic acid equivalents.

**Table 2:** Chemical characterization of the oil extracted from calafate pomace by supercritical CO_2_ and its comparison with fatty acids from other berries.

			Methyl	Esters (%)			
	Calafate Pomace	Maqui [51]	Murta [52]	Cranberry [48]	Blackberry [49]	Rosa Mosqueta [50]	Goldenberry [53]
C16:0	8.1 ± 0.8	9.1 ± 0.0	2.5 ± 0.3	3.7 ± 0.3	4.6 ± 0.4	3.1 ± 0.1	12.4 ± 0.1
C18:0	2.7 ± 0.1	3.0 ± 0.0	0.8 ± 0.1	1.3 ± 0.2	4.2 ± 0.1	1.9 ± 0.2	4.3 ± 0.0
C18:1	22.2 ± 0.7	38.3 ± 0.1	7.7 ± 0.2	15.9 ± 0.3	19.9 ± 0.5	14.3 ± 0.1	16.5 ± 0.1
C18:2ω6	30.0 ± 0.1	42.7 ± 0.0	88.2 ± 0.9	55.9 ± 0.3	58.6 ± 0.8	44.2 ± 0.1	63.2 ± 0.2
C18:3ω3	36.7 ± 0.2	0.9 ± 0.0	0.8 ± 0.7	22.8 ± 0.5	9.1 ± 0.3	31.7 ± 0.8	0.4 ± 0.0
FAS	10.8	12.1	3.3	5.0	8.8	5.0	16.7
MUFAS	22.2	38.3	7.7	15.9	19.9	14.3	16.5
PUFAS	66.7	43.6	89	78.7	81.4	75.9	63.6
ω6/ω3	1:1.2	1:0.0	1:0.0	1:0.4	1:0.1	1:0.7	1:0.0
		Physical-chemistry parameters					
	Calafate pomace	Blackberry [48]	Blackcurrant [48]	Blueberry [48]	Strawberry [48]	Grapeseed [53]	Golden berry [53]
YV	159 ± 1	148 ± 1	177 ± 4	167 ± 1	180 ± 1	127.5 ± 4.5	116.3
SV	176 ± 10	190 ± 1	190 ± 5	190 ± 1	194 ± 1	188 ± 11.3	Nd
	Calafate Pomace	Maqui [48]	Blackberry [54]	Cranberry [54]	Nut [55]	Grapeseed	Goldenberry
L*	0.9 ± 0.0	39.4 ± 0.8	10.1 ± 0.2	1.8 ± 0.1	93.68	_	_
a*	1.2 ± 0.0	−0.9 ± 0.0	7.7 ± 1.2	2.5 ± 0.2	−3.6 ± 0.0	_	_
b*	4.7 ± 0.0	10.1 ± 0.6	16.2 ± 0.3	3.0 ± 0.2	18.2 ± 0.0	_	_
**Color**	Dark yellow	Soft yellow	Dark red	Dark yellow	Bright yellow		
			**Quality Parameters**				
	**Calafate**	**Blueberry** [56]	**Cranberry** [56]	**Raspberry** [56]	**Nut** [55]	**Grapeseed**	**Goldenberry** [53]
	**Pomace**						
**PV**	8.6 ± 0.4	8.7 ± 0.0	7.3 ± 0.1	8.4 ± 0.0	1.2 ± 0.0	_	nd
**FFA**	0.6 ± 0.1	2.1 ± 0.0	1.7 ± 0.0	4.1 ± 0.0	0.4 ± 0.0	_	2.9

Data are expressed as the mean ± standard deviation (*n* = 3). YV= iodine value (g I_2_/100 g oil); SV= saponification value (mg KOH/g lipid); PV = peroxide value (milli-equivalent O_2_/Kg lipid); FFA = free fatty acid (g Oleic acid/100 g lipid).

**Table 3:** Tocopherol and tocotrienol content* of oil extracted from calafate pomace by supercritical CO_2_ and comparison with tocols from different berry seed oils.

			Tocols (µg/g Oil)				
	α−Τ	α−Τ3	γ−Τ	γ−Τ3	δ−Τ	δ−Τ3	Total
Calafate pomace	75.4 ± 3.8	127.7 ± 4.0	_	203.7 ± 10.5	_	_	406.4
Maqui [59]	169.3 ± 11.3	323.8 ± 20.3	56.7 ± 2.9	5.7 ± 1.0	13.5 ± 3.5	53.9 ± 7.4	622.9
Blackberry [60]	25.4 ± 6.5	_	1.311 ± 15.5	20.0 ± 1.7	31.7 ± 1.5	_	1.388
Blueberry [60]	4.4 ± 0.2	_	34.4 ± 0.1	330.4 ± 11.4	_	6.0 ± 1.0	375.2
Cranberry [60]	48.3 ± 4.5	152.7 ± 5.8	90.7 ± 2.1	1.235 ± 6.1	_	_	1.532
Rasberry [60]	407.0 ± 22.9	_	1.640 ± 86.9	7.2 ± 0.3	53.3 ± 3.2	_	2.112
Strawberry [60]	_	_	260.3 ± 13.7	_	20.0 ± 3.8	_	280.3

* Data are expressed as the mean ± standard deviation (*n* = 3). [60]. α-T, γ-T, δ-T; α−, γ−, δ- Tocopherols. α-T3, γ-T3, δ-T3; α−, γ−, δ− Tocotrienols.

## Data Availability

The data presented in this study are available on request from the corresponding author.
